# Absence of Internal Low Echoes Can Be an Important Diagnostic Clue for Intraductal Tubulopapillary Carcinomas of the Pancreas: A Case Report

**DOI:** 10.7759/cureus.94496

**Published:** 2025-10-13

**Authors:** Yurie Kitano, Shoji Oura

**Affiliations:** 1 Department of Surgery, Kishiwada Tokushukai Hospital, Kishiwada, JPN

**Keywords:** distinct margins, enhanced posterior echoes, intraductal tubulopapillary neoplasm, no internal low echoes, pancreas

## Abstract

Due to the low incidence of intraductal tubulopapillary neoplasms (ITPNs) of the pancreas, no typical images of them have been clarified. A 57-year-old man visited our hospital due to vomiting and abdominal pain. A blood test showed a high white blood cell count of 17,200/μL and a markedly elevated amylase level of 4,035 IU/L. CT showed swelling of the pancreatic head, which highly suggested a possible pancreatic head tumor. The patient received ulinastatin and antibiotic therapy for pancreatic head tumor-induced acute pancreatitis. An upper gastrointestinal endoscopy revealed a mass at the greater duodenal papilla. Endoscopic biopsy of the duodenal mass pathologically showed that the duodenal tumor was an adenoma. Endoscopic ultrasound showed common bile and main pancreatic duct dilatation and an oval mass with distinct margins, internal high echoes, and no posterior echo attenuation around the bile duct-pancreatic duct junction. Despite the lack of a definitive diagnosis of pathological malignancy, potential relapse of needle biopsy-induced acute pancreatitis made us treat the patient with pancreatic head and duodenal resection, followed by regional node dissection to avoid undertreatment. Postoperative pathological study clarified the duodenal mass to be an intestinal-type adenoma and showed that the solid mass in the pancreatic duct was 18 mm in size and consisted of cuboidal to columnar atypical cells growing mainly in a tubular fashion with severe dysplasia and no mucin production around the tumor cells. Immunostaining of the tumor cells showed MUC5AC, MUC2, trypsin, and BCL1 negativity, leading to the diagnosis of intraductal tubulopapillary carcinoma. The patient recovered uneventfully, was discharged on the 11th day after the operation, and is scheduled to be followed up on an outpatient basis. Diagnostic physicians should note that ITPNs have distinct margins, enhanced posterior echoes, and no internal low echoes due to their pathological structures. In addition, it is also important for diagnostic physicians to note that ITPN patients less often develop icterus due to both the mass softness and the lack of mucin production.

## Introduction

Pancreatic cancer initially causes no specific symptoms and is often detected at advanced stages due to its anatomical characteristics. Although pancreatic cancer patients ultimately present various symptoms such as abdominal pain and weight loss [1], they are often unable to undergo curative resection even at their first symptom onset, generally showing extremely dismal clinical outcomes. On the other hand, patients with pancreatic head cancer tend to develop icterus and are sometimes able to undergo curative surgery after some kind of biliary drainage.

Intraductal tubulopapillary neoplasms (ITPNs) of the pancreas account for fewer than 1% of exocrine pancreatic tumors and non-invasive pancreatic cancers, have either high-grade dysplasia or non-invasive cancer components, and generally show growth patterns of tubule-forming structures [2]. In other words, they have both no or least fibrous components, often observed in pancreatic cancers, and the mass softness. In addition, ITPNs do not have mucin, unlike intraductal papillary mucinous neoplasms (IPMNs) [3,4]. These findings, therefore, rarely make ITPNs to cause icterus even when located at the pancreas head and quasi completely obstruct the main bile duct.

Like in the diagnosis of other solid malignancies, CT, MRI, and ultrasound are the mainstays in the diagnosis of pancreatic tumors. In addition, endoscopic retrograde cholangiopancreatography [5] and endoscopic ultrasound (EUS) [6] evaluations are imperative for diagnostic physicians to harvest the tissue of the target lesion in the pancreas. Typical image findings of ITPNs, however, remain uncertain due to the extremely low incidence of ITPNs.

Here, we report a rare case of ITPN arising in the pancreatic head without icterus, which makes us speculate that ITPNs have no internal low echoes due to their pathological structures.

## Case presentation

**Figure 1:**
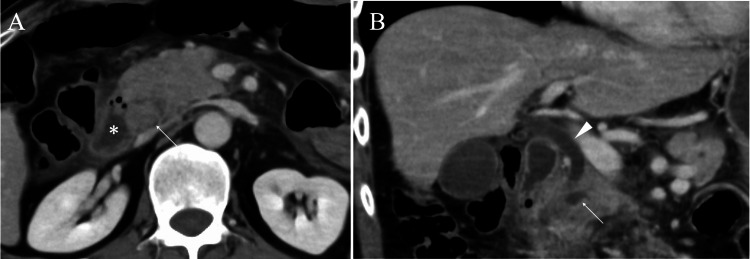
CT findings. (A) Axial view CT showing a presumed pancreatic head tumor (arrow) adjacent to the duodenum (asterisk). (B) Coronal view CT showing pancreatic (arrow) and bile (arrowhead) duct dilatation.

A 57-year-old man with intellectual disability visited our hospital due to vomiting and abdominal pain. Communication difficulties of the patient prevented us from determining the exact location of the abdominal pain. A blood test showed a high white blood cell count of 17,200/μL (reference range: 3,300-8,600/μL) and a markedly elevated amylase level of 4,035 IU/L (reference range: 44-132 U/L) with a 99.9% (reference range: 28-72%) pancreatic-type subunit. In addition, CT showed swelling of the pancreatic head, which highly suggested a possible pancreatic head tumor and made us speculate that the pancreatic head tumor could have caused acute pancreatitis (Figure 1).

**Figure 2:**
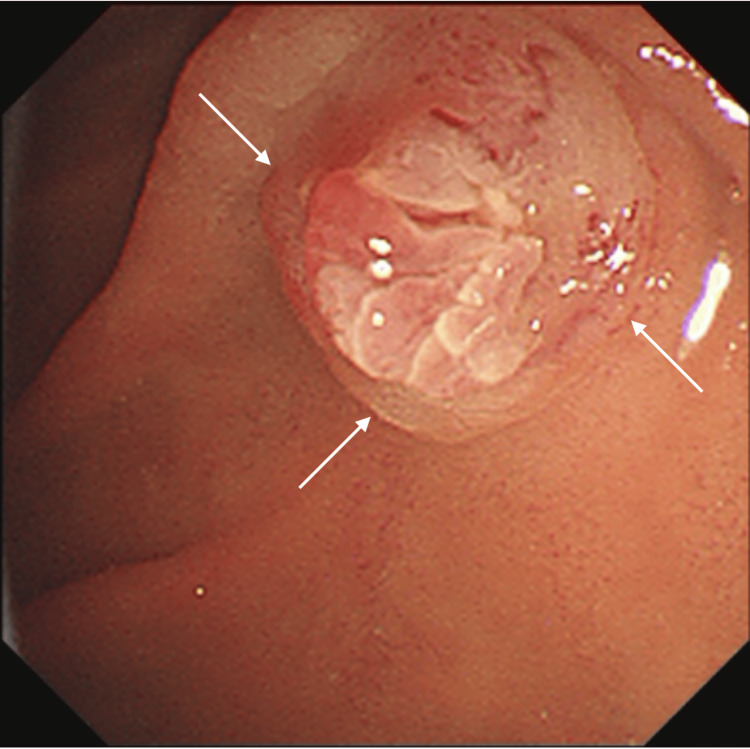
Endoscopic findings. An upper gastrointestinal endoscopy revealing a protruded mass (arrows) at the greater duodenal papilla.

The patient, therefore, received ulinastatin and antibiotic therapy for pancreatic head tumor-induced acute pancreatitis, leading to the prompt normalization of elevated amylase levels and symptom relief. An upper endoscopy performed to investigate abdominal pain revealed a mass in the greater duodenal papilla (Figure 2).

**Figure 3:**
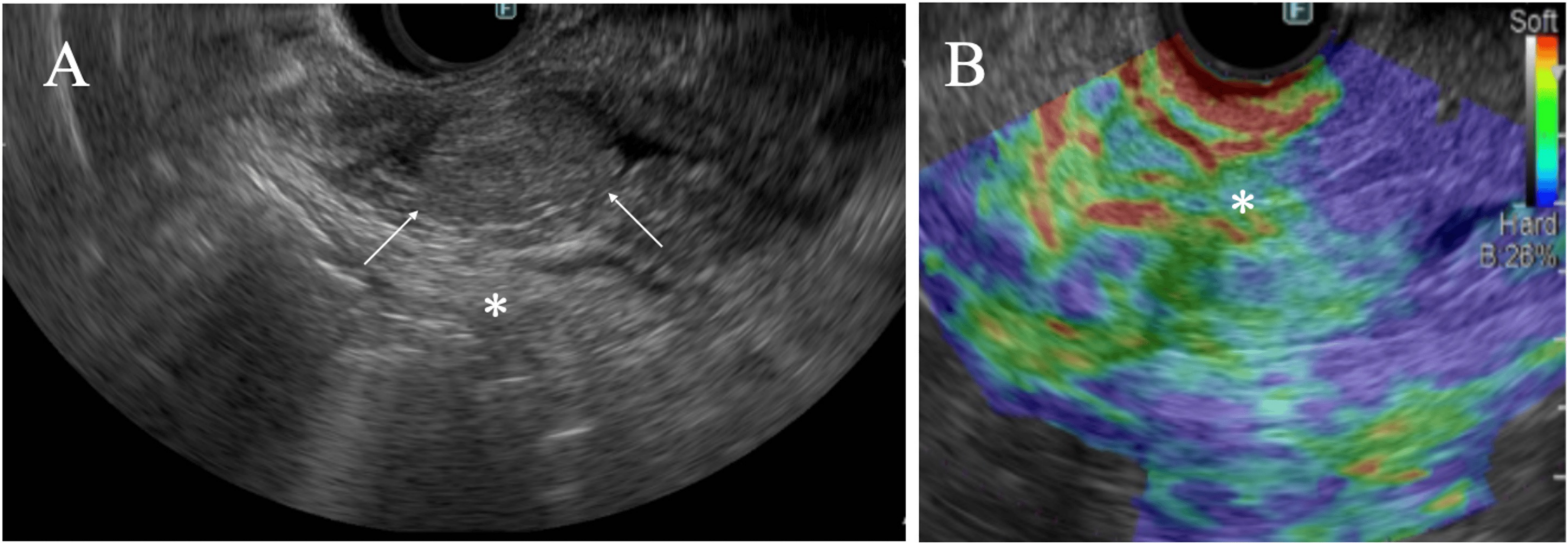
Endoscopic ultrasound (EUS) and elastography. (A) EUS showed a well-circumscribed mass (arrows) with internal high echoes and enhanced posterior echoes (asterisk). (B) Endoscopic elastography showed that the mass was expressed as greenish (asterisk).

Pathological study of the biopsy specimen showed atypical cells growing in a glandular fashion in the duodenal mucosa and led to the pathological diagnosis of a duodenal adenoma. EUS showed common bile and main pancreatic duct dilatation and an oval mass around the bile duct-pancreatic duct junction with distinct margins, internal high echoes, and no posterior echo attenuation, highly suggesting that the pancreatic head mass had caused acute pancreatitis and could be a possible malignancy (Figure 3).

**Figure 4:**
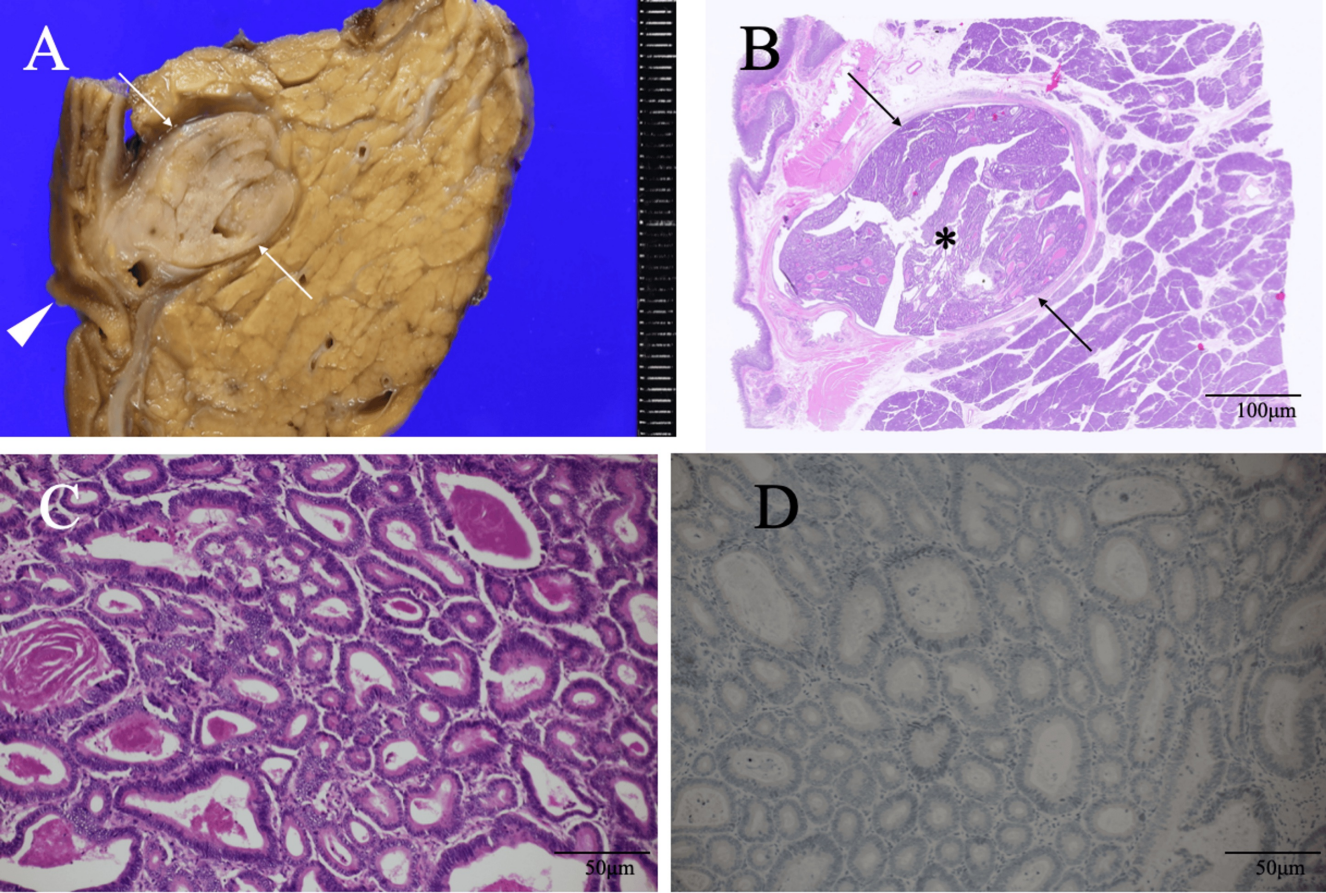
Pathological findings. (A) The bisected mass was a solid mass (arrows) with clear borders near the greater duodenal papilla (arrowhead). (B) Low magnified view showed that the pancreas head mass (asterisk) was seen occupying almost the entire dilated pancreatic duct (arrows). (C) Magnified view showed atypical cells growing in a tubular fashion. (D) MUC5AC immunostaining showed no positive cells.

Despite the lack of a definitive diagnosis of pathological malignancy, potential relapse of acute pancreatitis due to needle biopsy of the pancreatic head mass made us treat the patient with pancreatic head and duodenal resection, followed by regional node dissection to avoid undertreatment. Postoperative pathological study clarified the duodenal mass to be an intestinal-type adenoma and showed that the solid mass in the pancreatic duct was 18 mm in size and consisted of cuboidal to columnar atypical cells growing mainly in a tubular fashion with severe dysplasia and no mucin production around the tumor cells. Immunostaining of the tumor cells showed MUC5AC, MUC2, trypsin, and BCL1 negativity, leading to the diagnosis of intraductal tubulopapillary carcinoma (Figure 4).

The patient recovered uneventfully, was discharged on the 11th day after the operation, and is scheduled to be followed up on an outpatient basis.

## Discussion

Pancreatic cancer generally has abundant fibrous components, develops no symptoms until it becomes quite large or invades neighboring organs/tissues around the pancreas when located in the pancreatic body or tail, but causes bile duct and pancreatic duct stenosis or obstruction when located at the pancreatic head, sometimes leading to early detection of the pancreatic head cancer. In contrast, ITPNs only have delicate fibrovascular cores and lack fibrous component areas and are generally soft and well-demarcated tumors [2,7]. IPMNs also have no or least fibrous components but sometimes develop icterus when located at the pancreatic head and quasi obstruct the common bile duct [3,4]. Even if ITPNs are the same size and at the same location of pancreatic head IPMNs, they are less likely to cause icterus, presumably due to the mass softness and the lack of mucus.

Unlike icterus, ITPNs are prone to cause acute pancreatitis, as observed in this case. Diagnostic physicians, therefore, should strongly note that masses present in the pancreatic head, both with dilated bile and pancreatic ducts and no icterus, are highly ITPNs. In addition, when the target masses are judged as ITPNs on images and clinical findings, it is reasonable for attending surgeons to apply surgery to patients without pathological confirmation of the pancreatic head mass to avoid needle biopsy-induced pancreatitis.

It is well known that fibrous components not only give masses their hardness but also make mass margins unclear when present mixed with tumor cells at the tumor margins [8,9]. Conversely, ITPNs and IPMNs have no or least fibrous components at their mass margins, generally making their masses have well-circumscribed margins, a typical imaging finding.

EUS showed that the present ITPN had internal high echoes and enhanced posterior echoes. Internal high echos are formed through abundant ultrasound wave backscattering, which is generally caused by the presence of microvoids in papillary/tubular structures or of different acoustic impedance-bearing pathological components, e.g., fat [10]. ITPNs have tubulopapillary structures, including microvoids, and do not have areas of homogenous pathological components with similar acoustic impedance, which implies the lack of internal low echoes. In addition, fibrous components attenuate ultrasound waves, often causing posterior echo attenuation. ITPNs, however, have no or little fibrous components, which generally lead to making posterior echoes enhanced. IPMNs have similar pathological structures to those of ITPNs and naturally show predominant internal high echoes. IPMNs, however, always have mucinous areas within them and generally show at least focal low echoes or different internal high echoes to those caused by the papillary structures [11]. Therefore, it is imperative for diagnostic physicians to note that internal high echoes of IPMNs are slightly different from those of ITPNs (Table 1).

**Table 1:** Characteristics of ITPN, IPMN, ductal adenocarcinoma, and NET of the pancreas inferred by pathological constituents. ITPN: intraductal tubulopapillary neoplasm; IPMN: intraductal papillary mucinous neoplasm; NET: neuroendocrine tumor

Characteristics	ITPN	IPMN	Adenocarcinoma	NET
Icterus	Rare	Possible	Possible	Possible
Mucin production	-	++	±	±
Echo findings				
Internal low echoes	None	Focally present	Frequent	Frequent
Posterior echoes	Enhanced	Enhanced	Attenuated	Enhanced

Although various etiologies of ampullary adenomas have been suggested, one of which is irritation by the bile [12]. Why an ampullary adenoma and an ITPN had coexisted closely remains naturally uncertain. However, it cannot be denied that the presence of ITPN in the pancreatic head may have increased the stimulation to the papilla, resulting in the development of the ampullary adenoma.

Well-defined tumors in the pancreas head, accompanied by bile duct and pancreatic duct dilation, with internal high echoes and enhanced posterior echoes, are likely to be ITPNs or IPMNs. In addition, when lacking icterus despite the presence of main bile duct dilatation, diagnostic physicians should suspect that the pancreatic head tumors are more likely to be ITPNs than IPMNs.

## Conclusions

We highly speculate that both ITPNs and IPMNs can be located at the pancreatic head and show distinct margins due to the lack of fibrous components at mass borders. Diagnostic physicians should include ITPNs and IPMNs in the differential diagnosis of pancreatic head tumors when tumors have clear margins, no internal low echoes, and enhanced posterior echoes. In addition, when pancreatic head tumors have dilatation of the bile and pancreatic ducts and no icterus, physicians should strongly suspect them to be ITPNs.
